# Preface to the Journal of Dermatology special issue: The changing landscape of melanoma treatment

**DOI:** 10.1111/1346-8138.17146

**Published:** 2024-02-15

**Authors:** Yasuhiro Fujisawa

**Affiliations:** ^1^ Ehime University Dermatology To‐on Japan

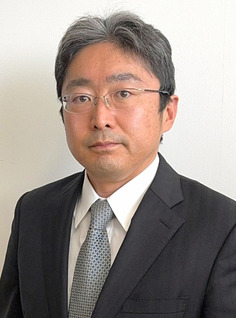

Malignant melanoma historically considered one of the most life‐threatening tumors, poses significant challenges regarding treatment due to the lack of effective systemic therapies. Before the introduction of immune checkpoint inhibitors and BRAF‐inhibitors, radical surgery to prevent further spread was believed to be the best option for the resectable disease. Thus, a 5‐cm wide horizontal margin was widely accepted. In some cases, more aggressive surgery, such as amputation, was considered. Prophylactic lymph node dissection was also regarded a standard of care. However, such a radical surgical approach had limitations as many patients developed metastases and died of the disease.

Recognizing the need for improved outcomes and reduced adverse events, numerous clinical trials were initiated. These trials aimed not only to enhance treatment effectiveness, but also to minimize the impact of adverse events, marking a pivotal shift in the quest for more advanced and tailored melanoma therapies.

In the context of this evolution, in this special issue, the article by Koizumi et al. reviews the changes in surgical approaches to melanoma treatment, noting a trend toward “smaller” operations. Additionally, Uchi et al. delve into systemic therapy options, discussing new treatment modalities such as immune checkpoint inhibitors and BRAF inhibitors. As Fukushima et al. in their article review the development of effective systemic therapy in the advanced disease that has proved to be useful as adjuvant therapy, further extending the impact of these advancements.

Namikawa et al. focus on patients experiencing long‐lasting effects of such therapy and discuss promising new agents currently in development.

There is no doubt that these new therapeutic options have delivered higher efficacy and longer survival compared to decades ago, making this a transformative era in the management of advanced disease.

I believe that these reviews contribute to a clearer understanding and will inspire young dermatologists to explore and specialize in melanoma treatment.

